# Airborne pollen and fungal spores in Garki, Abuja (North-Central Nigeria)

**DOI:** 10.1007/s10453-016-9443-5

**Published:** 2016-05-20

**Authors:** Dimphna Nneka Ezike, Catherine V. Nnamani, Oluwatoyin T. Ogundipe, Olushola H. Adekanmbi

**Affiliations:** 1 Department of Applied Biology, Faculty of Science, Ebonyi State University, Abakaliki, Ebonyi Nigeria; 2 Department of Botany, Faculty of Science, University of Lagos, Akoka, Lagos Nigeria

**Keywords:** Abuja, Allergy, Atmosphere, Meteorological parameters, Pollen, Fungal spores

## Abstract

The ambient atmosphere is dominated with pollen and spores, which trigger allergic reactions and diseases and impact negatively on human health. A survey of pollen and fungal spores constituents of the atmosphere of Garki, Abuja (North-Central Nigeria) was carried out for 1 year (June 1, 2011–May 31, 2012). The aim of the study was to determine the prevalence and abundance of pollen and fungal spores in the atmosphere and their relationship with meteorological parameters. Airborne samples were trapped using modified Tauber-like pollen trap, and the recipient solutions were subjected to acetolysis. Results revealed the abundance of fungal spores, pollen, fern spores, algal cysts and diatoms in decreasing order of dominance. The atmosphere was qualitatively and quantitatively dominated by pollen during the period of late rainy/harmattan season than the rainy season. Numerous fungal spores were trapped throughout the sampling periods among which *Alternaria* spp., *Fusarium* spp., *Cladosporium* spp. and *Curvularia* spp. dominated. These fungi have been implicated in allergic diseases and are dermatophytic, causing diverse skin diseases. Other pathogenic fungi found in the studied aeroflora *were Dreschlera* spp., *Helminthosporium* spp., *Torula* spp., *Pithomyces* spp., *Tetraploa* spp., *Nigrospora* ssp., *Spadicoides* spp., *Puccinia* spp*. and Erysiphe graminis*. Total pollen and fungal spores counts do not show significant correlation with meteorological parameters.

## Introduction

Pollen and fungal spores are the most dominant aeroallergens, because of their ubiquitous and wide distribution in time and space than any other representatives of living matter (Shahali et al. 2007). Their dispersal in the atmosphere is modulated by meteorological parameters such as rainfall, humidity, temperature, wind velocity and strength. Geography and vegetation also play a crucial role in the type of pollen or spores present in the atmosphere of any region (Burge 2002; Perveen et al. 2007). The clinical significance of airborne particles is largely related to their allergenicity which is influenced by their numbers and perhaps their bulk concentrations. Allergies due to pollen are seasonal, and hypersensitive individuals living in an area with high atmospheric concentration of anemophilous plants have a higher risk of developing allergic sensitization. Atmospheric pollen and fungal spores are recognized to provoke allergic sensitizations such as conjunctivitis, extrinsic rhinitis and asthma. Their importance in clinical allergy has been well established for many years ago (Li and Kendrick 1995). The provocation of pollen allergies is due to allergen protein contained in the sporoderm and cytoplasm (Perveen et al. 2007). Knowledge of their prevalence is required for a rational approach to diagnosis and management of allergic diseases (Chatterjee and Hargreave 1974).

Airborne fungal spores’ concentration could be used as an indicator of pathogen development and could be useful when the infection levels are initially determined by inoculum rather than the weather. In these conditions, monitoring of airborne inoculums and their relationship with meteorological data provides a valuable tool for establishing the basis for an accurate modern integrated pest management strategy (Escuredo et al. 2011).

Airborne pollen have proved to be extremely relevant in the evaluation of the vegetation characteristics of a study area, understanding of the flowering periodicity and in developing a functional model for predicting pollen concentration in the atmosphere. Most aeropalynological works have been carried out in Nsukka (South-East Nigeria) (Agwu and Osibe 1992; Agwu 1997; Njokuocha 2006), in Lagos (South-West Nigeria) (Adeniyi et al. 2014; Adekanbi and Ogundipe 2010) in Rivers State (South-south) (Agwu and Osibe 1992). The present study was carried out in Garki, Abuja, over a period of 12 months. The objectives of the study were to ascertain (a) the atmospheric pollen and fungal spores (aeroallergens) concentration in Garki, and (b) the seasonal prevalence of airborne pollen and fungal spores and their relationship to meteorological parameters.

## Materials and methods

### Study area

**Fig. 1:**
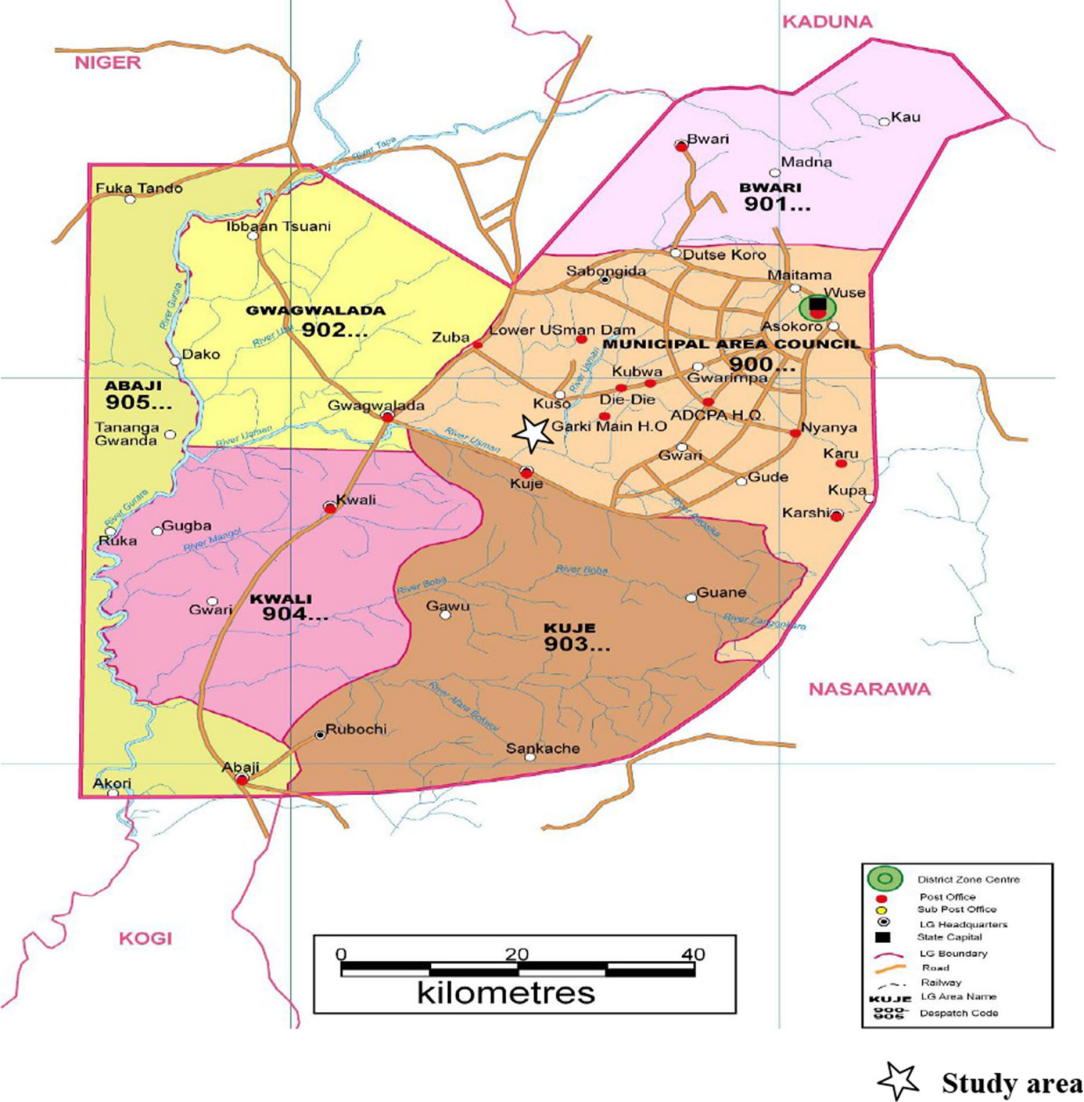
Map of Abuja (North-Central Nigeria), showing the study area

The study was conducted in Garki, Abuja, the Federal Capital Territory of Nigeria, located north of the confluence of the River Niger and Benue River. The Federal Capital Territory has an area of 7314.2 km^2^, and the actual city occupies 273.3 km^2^. It is bordered by Niger state to the West and North, Kaduna to the North-East, Nasarawa to the East and South and Kogi to the South-West (Fig. 1).

The climate is influenced by two main seasons: the rainy season which lasts from May to September and dry season from October to April. The area belongs to the tropical region with average monthly temperature fluctuating between 24.7 and 34.3 °C. The mean annual rainfall varies from 19.0 to 130.3 mm. The vegetation is a mosaic of lowland Rainforest and secondary Grassland (Ofomata 1975; White 1983). The sampled site is located at 9°0′0″N and 7°30′3″E. Grassland dominates the vegetation around the trap and is characterized by *Panicum maximum*, *Andropogon tectorum*, *Imperata cylindrica*, *Anthephora ampulacea*, *Imperata cylindrica*, *Pennisetum purpureum* and *Hyparrhenia barteri*. The vegetation is highly diverse and includes lowland Rainforest taxa in protected areas and derived Savanna dominated by *Elaeis guineensis*, *Alchornea cordiforlia*, *Pentacletra macrophylla*, *Gloriosa superba*, etc. The herbaceous plants were dominated by *Aspilia africana* and *Ageratum conyzoides*.

### Sample collection

**Fig. 2:**
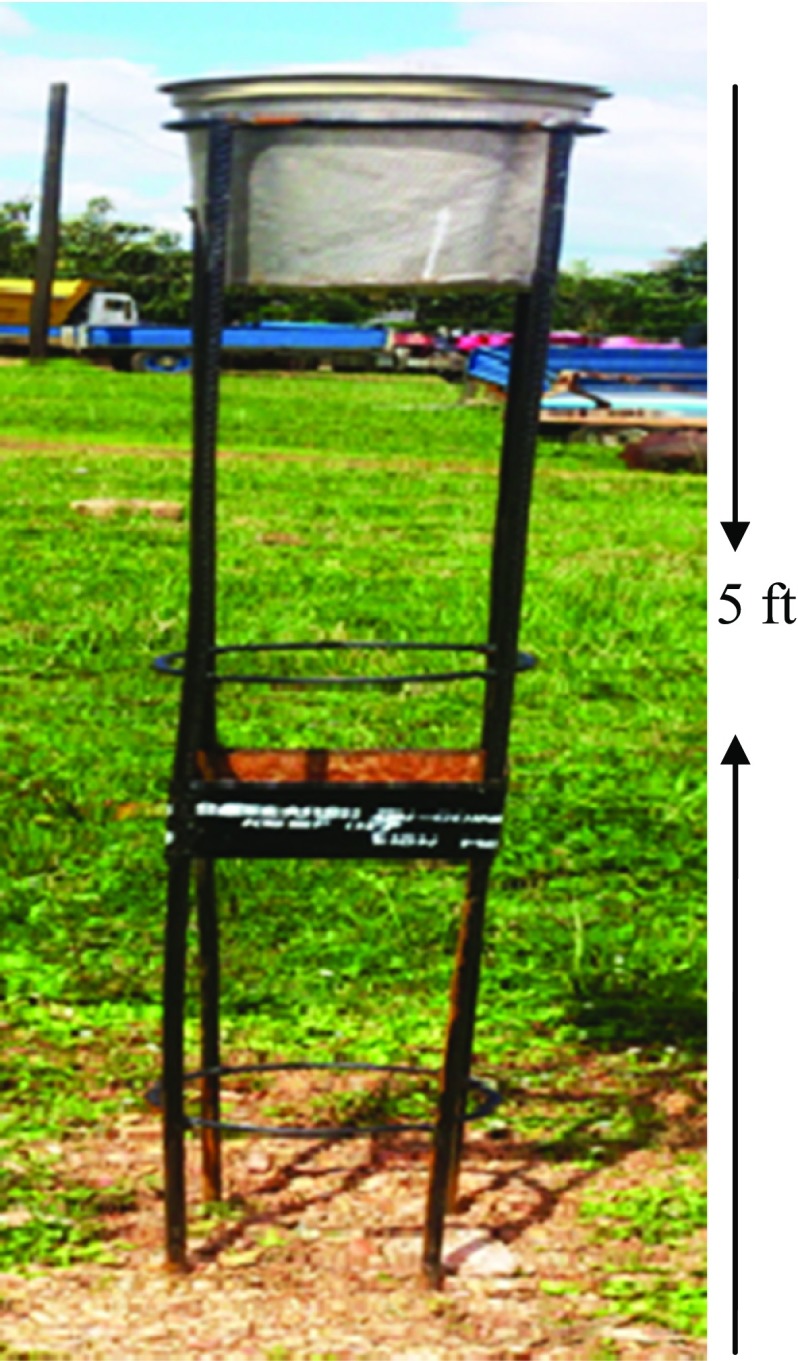
Modified Tauber-like pollen trap in the field

Modified Tauber-like pollen trap was employed for the collection of the airborne pollen and spores. The trap was placed at the height of 5 ft above the ground level (Fig. 2). A solution made of glycerol (50 ml), formaldehyde (10 ml) and phenol (5 ml) was prepared and poured into the trap. The recipient solutions were collected monthly for the period of 1 year. Samples were sieved through 200-µm mesh wire gauze to filter off large organic particles. The liquid with suspended palynomorphs was centrifuged at 2500 revolution per minute for 5 min to recover the palynomorphs residues. The residues were washed three times with water and were acetolyzed according to a modified version of Erdtman (1971) procedures; acetolysis mixture which consists of concentrated sulfuric acid and acetic anhydride in the ratio of 9: 1 was prepared; 5 ml of the acetolysis mixture was poured into each sample and placed in water bath for 10 min at 100 °C. They were centrifuged, decanted and washed twice with distilled water. The recovered residues were stored in vials with two drops of glycerine. Temporary slides were prepared and examined using light Olympus CH Trinocular microscope (LM), equipped with 650 IS Cannon Digital camera at 400× and 100× magnifications. Identification was based on comparison with reference collection of pollen slides, description and photomicrographs of pollen and spores using books and journals (Agwu and Akanbi 1985; Y’bert 1979). Agwu (1997) and Agwu and Osibe (1992) also used Tauber-like pollen traps similar to the trap employed in this study and also the same methodology.

Pollen and fungal spores obtained were counted monthly and expressed in frequency. The data obtained were analyzed using the SPSS statistical package version 20 (SPSS Inc. Chicago, Illinois USA). Correlation coefficients were generated to examine the relationship among pollen, fungal spores frequency and meteorological data. Meteorological data were obtained from Nigerian Meteorological Centre Abuja, Nigeria.

## Results

**Fig. 3:**
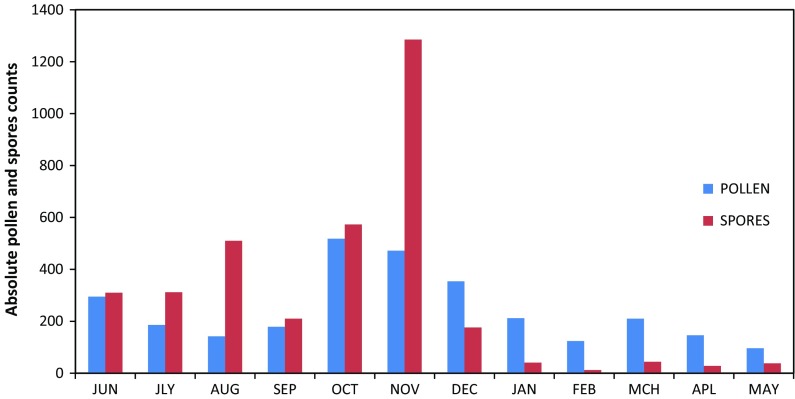
Monthly variations of atmospheric pollen and fungal spores from June 2011 to May 2012 in Garki

Fifty-three pollen types belonging to 36 families were identified (Table 1). Three, thirteen and 37 pollen were identified to family, generic and species levels, respectively. The atmosphere was qualitatively and quantitatively dominated with varied species of fungal spores. Eighteen fungal spore types were identified (Table 2). The annual contribution of fungal spores of 3534 (54.98 %) was found to be greater than the 2852 pollen grains (45.01 %). The months with the highest pollen abundance were October 518 (17.65 %), November 472 (16.08 %) and December 354 (12.07 %). May 96 (3.37 %) and June 94 (3.29 %) had the lowest pollen records (Fig. 3). High values of fungal spores were recorded in November, which was essentially dominated by *Erysiphe graminis* accounting 93 % of its annual total. February with 12 spores (0.34 %) had the lowest record of fungal spores (Fig. 2; Table 2). The major pollen contributors were Poaceae 524 (18.4)*, Pentaclethra macrophylla* 402 (14.1)*, Elaeis guineensis* 365(12.80), *Justicia* spp. 211 (7.39), *Cassia* spp. 160 (5.6), *Alchornea cordiforlia* 103 (3.61) and *Luffa* spp. 102 (3.57).

**Table 1:** Frequency of atmospheric pollen of Garki, Abuja (North-Central Nigeria), from June 2011 to May 2012

S. no	Pollen	Months													
		Jun	Jul	Aug	Sep	Oct	Nov	Dec	Jan	Feb	Mar	Apr	May	Total	%
1	*Acacia* spp.	2	0	2	2	0	0	0	0	0	0	0	0	6	0.21
2	*Adansonia digitata*	0	0	0	0	0	12	4	0	0	0	0	0	16	0.56
3	*Ageratum conyzoides*	0	0	0	0	12	0	0	16	0	0	0	0	28	0.98
4	*Albizia* spp.	0	2	0	0	0	0	0	0	0	0	0	0	2	0.07
5	*Alchornea cordiforlia*	1	0	0	12	2	52	8	2	16	4	6	0	103	3.61
6	*Aloe bateri*	0	0	0	0	3	0	0	0	0	0	0	0	3	0.11
7	*Amaranthaceae/Chenopodiaceae*	0	0	44	8	0	16	0	4	0	0	0	0	72	2.52
8	*Aneilema beninlense*	0	2	0	0	0	0	0	0	0	0	0	0	2	0.07
9	*Anthocleista djalonensis*	0	0	0	4	0	0	0	20	0	0	0	0	24	0.84
10	*Aspilia africana*	0	0	6	22	2	8	0	0	0	0	1	6	45	1.58
11	*Asteraceae*	4	0	0	0	0	0	0	0	0	0	0	0	4	0.14
12	*Bridelia ferruginea*	0	0	0	0	24	0	0	0	0	0	0	0	24	0.8
13	*Bulbostylis filamentosa*	0	0	0	0	0	0	4	0	0	0	0	0	4	0.14
14	*Cassia* spp.	28	0	0	4	10	8	0	0	8	30	16	56	160	5.6
15	*Casuarina equisetifolia*	1	6	2	0	0	0	0	0	0	8	6	4	27	0.95
16	*Ceiba pentandra*	0	4	0	0	0	0	0	0	0	0	0	0	4	0.14
17	*Celtis zenkeri*	0	0	0	0	0	16	20	0	0	0	0	0	36	1.26
18	*Cissus* spp.	0	0	0	2	0	0	0	0	0	0	0	0	2	0.1
19	*Cochlospermum tinctorum*	0	16	0	0	0	0	42	0	0	0	0	0	58	2.03
20	*Cocos nucifera*	4	0	0	0	0	0	0	0	0	0	0	0	4	0.14
21	*Crotalaria* spp.	0	0	0	0	0	0	0	6	4	0	0	0	10	0.35
22	*Cyperus esculenta*	0	0	0	4	0	44	4	1	10	6	2	2	73	2.56
23	*Dichrostachys cinera*	0	0	0	0	0	0		2	8	0	0	0	10	0.35
24	*Dracaena arborea*	0	0	8	0	2	0	0	0	0	0	0	0	10	0.35
25	*Drypetes gilgiana*	0	0	0	0	0	0	2	0	0	0	0	0	2	0.07
26	*Eichlornia natans*	2	0	0	0	0	0	0	0	0	0	0	0	2	0.07
27	*Elaeis guineensis*	118	6	8	5	6	206	8	8	8	0	0	0	365	12.80
28	*Eugenia* spp.	0	0	0	0	2	0	0	0	0	0	0	0	2	0.07
29	*Gardenia imperialis*	0	0	0	0	0	0	2	0	0	0	0	0	2	0.07
30	*Gloriosa superba*	0	0	2	0	32	0	2	6	0	0	0	0	42	1.50
31	*Hexabolus scrispiflorus*	0	0	0	0	0	0	8	0	0	0	0	0	8	0.28
32	*Hymenocardia acida*	0	2	4	12	12	0	0	0	6	0	20	0	56	1.96
33	*Ipomea* spp.	0	0	0	0	0	0	0	8	32	0	0	0	48	1.68
34	*Justicia* spp.	0	8	8	0	0	2	4	13	18	134	14	12	213	7.47
35	*Khaya senegalensis*	0	0	2	30	18	48	0	0	0	0	0	0	98	3.44
36	*Lannea acida*	63	6	2	0	0	0	0	0	0	0	0	0	71	2.40
37	*Lannea welwitschii*	0	0	2	0	0	0	0	0	0	0	0	0	2	0.07
38	*Lophira alata*	0	0	0	0	2	0	0	0	0	0	0	0	2	0.07
39	*Luffa* spp.	0	0	0	0	21	2	0	6	12	26	73	2	102	3.58
40	*Marantochloa cuspidate*	0	0	2	0	0	0	0	0	0	0	0	0	2	0.07
41	*Milletia* spp.	2	0	0	0	0	0	0	0	0	0	0	0	2	0.07
42	*Olax subscorpoides*	0	0	0	0	0	0	12	12	0	0	0	0	24	0.84
43	*Parkia biglobosa*	2	0	0	0	6	0	0	16	0	0	0	0	24	0.84
44	*Pentaclethra macrophylla*	0	0	4	120	0	36	188	54	0	0	0	0	402	14.10
45	Poaceae	48	44	44	68	248	20	28	16	8	0	0	0	524	18.37
46	*Phyllanthus discoides*	2	0	0	0	0	0	0	0	0	0	0	0	2	0.07
47	*Polygala multiflora*	0	0	2	0	0	0	2	0	0	0	0	0	4	0.14
48	*Solenostemon monostachys*	0	0	0	0	0	0	0	2	0	0	0	0	2	0.07
49	*Syzygium* spp.	0	0	0	0	0	0	12	2	0	0	0	0	14	0.50
50	*Uapaca togoensis*	14	0	0	0	4	0	0	0	2	2	8	14	44	1.54
51	Terminalia spp.	4	0	0	0	0	0	2	0	0	0	0	0	6	0.21
52	Vernonia spp.	0	0	0	0	0	0	0	12	0	0	0	0	12	0.42
53	*Vigna multinervis*	0	0	0	0	0	2	0	0	0	0	0	0	2	0.07
	Total pollen	294	94	142	181	518	472	354	212	124	210	146	96	2852

**Table 2:** Frequency of atmospheric fungal spores of Garki, Abuja (North-Central Nigeria), from June 2011 to May 2012

S. no	Fungal spores	Months													
		Jun	Jul	Aug	Sep	Oct	Nov	Dec	Jan	Feb	Mar	Apr	May	Total	%
1	*Alternaria* spp.	14	4	0	6	0	0	0	0	0	0	18	28	70	1.98
2	*Apiosporina* spp.	0	0	0	0	2	0	0	0	0	0	0	0	2	0.06
3	*Aspergillus* spp.	0	0	2	0	0	0	1	0	0	0	0	0	3	0.08
4	*Cercosporella* spp.	3	0	0	0	0	0	0	0	0	0	0	0	3	0.08
5	*Cladosporium* spp.	15	24	0	0	45	0	8	4	0	0	0	0	96	2.72
6	*Curvularia* spp.	0	4	12	9	96	2	0	18	12	0	0	0	153	4.33
7	*Erysiphe graminis*	0	0	2	0	0	1200	24	9	0	0	0	0	1235	34.95
8	*Fusarium* spp.	0	0	6	0	0	20	0	0	0	0	0	0	26	0.74
9	*Hansfordiella* spp.	37	123	90	6	64	0	0	0	0	0	0	0	320	9.05
10	*Helminthosporium* sp.	6	0	6	30	0	6	8	0	0	0	0	0	56	1.58
11	*Pithomyces* spp.	12	15	20	20	54	6	36	0	0	0	0	0	163	4.61
12	*Nigrospora* spp.	51	6	48	12	45	12	0	6	0	24	10	10	224	6.34
13	*Puccinia* spp.	12	28	189	60	3	1	1	0	0	0	0	0	294	8.32
14	*Spadicoides* spp.	0	75	0	30	46	0	0	0	0	20	0	0	171	4.84
15	*Sporidesmium* spp.	32	0	0	0	165	0	0	0	0	0	0	0	197	5.57
16	*Tetraploa* spp.	111	3	9	3	2	0	12	0	0	0	0	0	140	3.96
17	*Torula* spp.	15	28	6	12	3	1	1	0	0	0	0	0	66	1.87
18	*Venturia* spp.	0	0	0	1	8	0	0	0	0	0	0	0	9	0.25
19	Indeterminate	2	2	120	21	40	24	84	0	0	0	0	0	293	8.29
	Total pollen	310	312	510	210	573	1285	175	37	12	44	28	38	3534

**Fig. 4:**
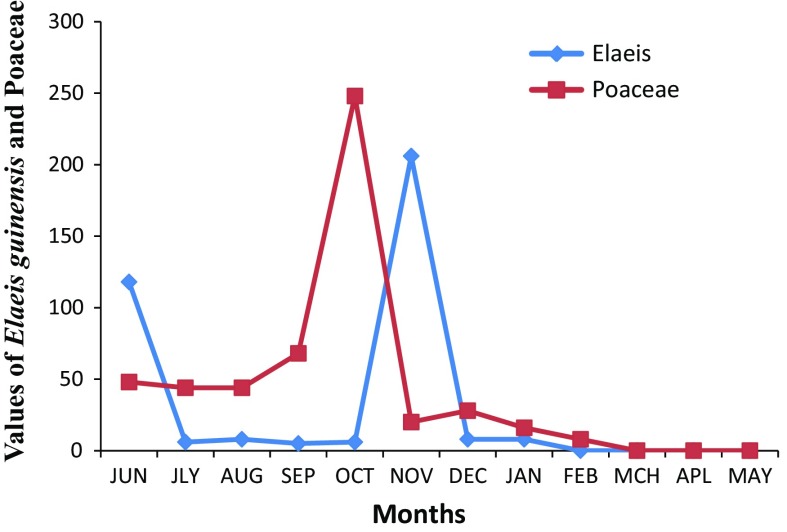
Monthly variations of Poaceae and *Elaeis guineensis* pollen from June 2011 to May 2012 in Garki

The anemophilous pollen recorded from aeroflora of Abuja include those of Poaceae, *Elaies guineensis*, *Cyperus esculenta*, Amaranthaceae/Chenopodiaceae, *Terminalia* sp., *Casuarina equisetifolia*, *Cocos nucifera* and *Dracaena arborea*. The overall percentage contribution of anemophilous pollen (71 %) was higher than recorded by entomophilous pollen (29 %). Anemophilous pollen dominated from the month of June to February, while the entomophilous were more abundant from the month of September to May. Poaceae and *Elaeis guineensis* were the most pollen producers taxa. Poaceae pollen showed the maximum abundance in October; *Elaeis guinensis* pollen delayed 1 month and peaked in November (Fig. 4).

Airborne pollen grains were grouped into three categories based on their sources: trees/shrubs, herbs and grasses pollen. Trees and shrubs comprised of 51.61 %, grasses and herbs reached 32.70 and 15.68 %, respectively. Trees and shrubs pollen were the bulk contributors of the atmospheric pollen, and their dominance could be demarcated between the months of September to February. The dominant trees and shrubs pollen at the studied location were: *Pentaclethra macrophylla* 402 (14.10)*, Elaeis guineensis* 365 (12.80), *Justicia* spp. 213 (7.47), *Lannea acida* 71 (2.4), *Alchornea cordiforlia* 103 (3.61) and *Khaya senegalensis* 98 (3.44) among others.

As regards the fungal spores, the atmosphere was dominated by different species such as *Erysiphe graminis* 1235 (34.95 %)*, Hansfordiella* spp. 3209 (9.05), *Puccinia* spp. 294 (8.32), *Nigrospora* spp. 224 (6.34), *Pithomyces* spp. 163 (4.61) and *Curvularia* spp. 153 (4.33). Most spores were more abundant from the months of June to December, and their atmospheric load declined after the month of December.

**Fig. 5:**
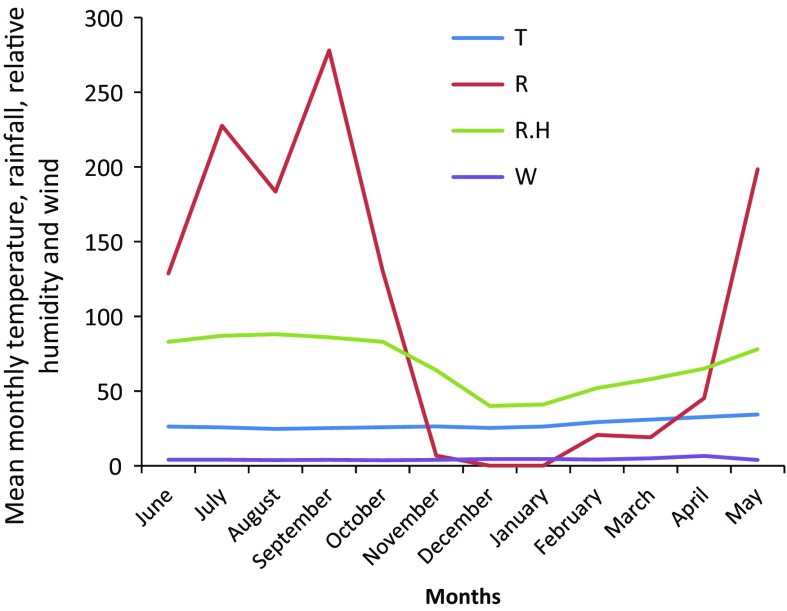
Mean monthly values of meteorological parameters in Garki (June 2011–May 2012). Mean monthly values of meteorological parameters in Garki (June 2011–May 2012). *T* Mean monthly temperature (°C), *R* mean monthly rainfall (mm); *R.H.* mean monthly relative humidity (%), *W*, mean monthly wind speed (km/h)

**Fig. 6:**
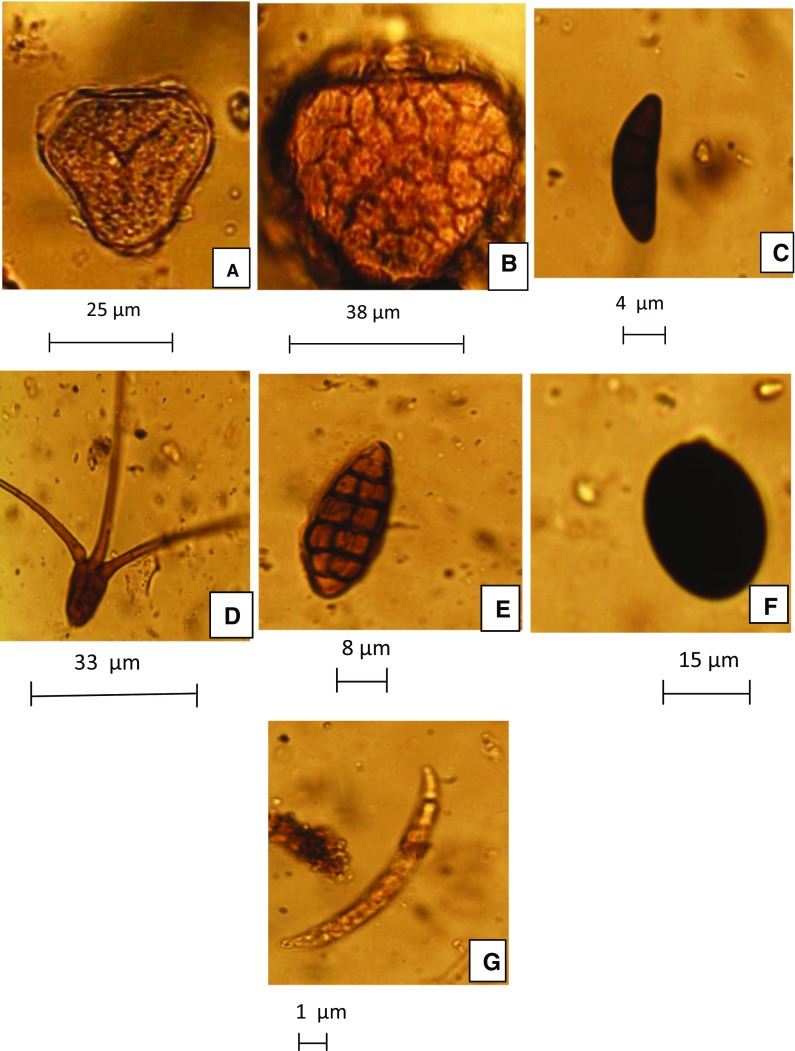
Photomicrographs of some recovered pollen and fungal spores in the atmosphere of Garki. Pollen **a** *Elaeis guineensis*, **b** *Bombax buonopozense*, fungal spores **c** *Curvularia* sp.; **d** *Tridentarium* sp.; **e** *Alternaria* sp.; **f** *Nigrospora* sp.; **g**, *Fusarium* sp. All magnifications ×400

The relationship between pollen counts and weather parameters was correlated (Fig. 5). The total pollen counts correlated not significantly, negatively with rainfall, relative humidity and wind but positively with temperature. Poaceae pollen concentration was positively correlated with rainfall and humidity, whereas it was negatively correlated with increasing temperature and wind. The pollen of *Luffa* sp. showed a positive and highly significant coefficient of correlation with wind (Table 3). There was a positive but not significant correlation between the total fungal spores count and relative humidity (Table 4). However, most dominant fungal spores with the exception of *Erysiphe graminis* correlated positively with rainfall and relative humidity and negatively with the wind (Table 4). *Puccinia* spp. showed a negative and highly significant coefficient of correlation with temperature. *Hansfordiella* spp. was associated positively and significantly with relative humidity (Table 4). The photomicrographs of some recovered pollen and fungal spores are shown in Fig. 6.

**Table 3:** Correlation coefficients between frequency of pollen and meteorological parameters

Pollen count	*R*	*T*	R.H	*W*
*Alchornea cordiforlia*	−.466	.111	−225	−.106
*Cyperus* spp.	−.434	.109	−.293	.334
*Elaeis guineensis*	−.237	.004	.044	−.213
*Justicia* spp.	.346	−.273	−.293	.334
*Luffa* spp.	−.186	.347	−.132	.917**
*Pentaclethra macrophylla*	−.371	−.016	−.443	−.120
Poaceae	.289	−.162	.415	−.411
Total pollen count	−.352	.019	−.176	−.243

** Correlation is significant at *p* = 0.01 level (2-tailed)

*R* mean monthly rainfall (mm), *T* mean monthly temperature (°C), *R.H* mean monthly relative humidity (%), *W* mean monthly wind speed (km/h)

**Table 4:** Correlation coefficients between frequency of fungal spores and meteorological parameters

Spores count	R	T	R.H	W
*Curvularia* spp.	.087	−.087	.211	−.323
*Erysiphe graminis*	−.312	.022	−.097	−.146
*Hansfordiella* spp.	.555	−.558	.654*	−.373
*Nigrospora* spp.	.279	−.507	.590	−.291
*Pithomyces* spp.	.236	−.317	.262	−.400
*Puccinia* spp.	.485	−.933**	.497	−.298
*Spadicoides* spp.	.550	.067	.499	−.246
*Sporidesmium* spp.	.100	.010	.299	−.286
*Tridentarium* spp.	.094	−.058	.249	−.133
Total fungal spores	−.021	−.324	.266	−.405

** Correlation is significant at the *p* = 0.01 level (2-tailed)

* Correlation is significant at the *p* = 0.05 level (2-tailed)

*R* mean monthly rainfall (mm), *T* mean monthly temperature (°C), *R.H.* mean monthly relative humidity (%), *W* mean monthly wind speed (km/h)

## Discussion

The results provided detailed information about the relative abundance of the source plants as well as their pollination periods. The number of pollen types (53) recorded in this work for 1 year is higher compared to 40 pollen types belonging to twenty-six families recovered in 1 year in two sampling sites in Nsukka plateau, South-East Nigeria (Njokuocha 2006). Thirty-seven pollen types belong to 30 families in Shomolu Local Government Area, South-West Nigeria (Adeniyi et al. 2014). The vegetation was therefore inferred rich and more diverse than previous studied areas in Nigeria. Flowering was observed throughout the year though with some variations. The dominant trees, shrubs and grass pollen at the studied location reflect the mosaic of lowland Rainforest and secondary Grassland ecotype as indicated by Ofomata (1975) and White (1983). This is consistent with the work of Nnamani and Uguru (2013), who reported the abundance of these species in their work carried out in Nigeria. The atmosphere contained more pollen during the dry season (October to March), especially at harmattan period (October to January) than the rainy season (April to September). Major pollen contributors during the rainy season were Poaceae and *Elaeis guineensis,* and they reached their monthly maximum load in dry season. Large recovery of *Elaeis guineensis* pollen could reflect the extent of palm forest within the study area. More pollen dominated the atmosphere at dry and more windy periods than the rainy and humid periods. Harmattan period (October to January) was designated as a higher risk period for hypersensitive individuals to pollen. The greater intensity of rainfall inferred through its amount and duration leads to a decline in pollen morphotypes, since rainfall washes down suspended pollen in the atmosphere.

Among the anemophilous pollen, Poaceae and *Elaeis guinensis* pollen were the most abundant. Njokuocha (2006) and Agwu and Osibe (1992) also found them preponderant in their studies. Poaceae pollen include the wild and cultivated grass pollen, which were more abundant from June and December; pollen allergies at this period could be attributed partly to their higher antigenic load. Taketomi et al. (2008) founded the pollen of Poaceae family as a major sensitizing agent in patients with pollinosis. D’Amato et al. (2007) stated also that grass-induced pollinosis is the most common pollen allergy in Europe.

Anemophilous pollen have been reported to be more implicated in allergies because of their higher atmospheric load. Chatterjee and Hargreave (1974) stated that they also have better aerodynamic properties than the entomophilous pollen. Furthermore, anemophilous plants produce more pollen than entomophilous species as their pollination is hazardous. This could have led to their more preponderance than the latter. Their smaller sizes make them easily trapped on skin, conjunctiva of the eyes and mucous membrane of the nose; they might penetrate the lower respiratory tract and induce symptoms of bronchial asthma and/or hay fever (Abou Chakra et al. 2009).

The dominance of *Erysiphe graminis* coincided with the decline of Poaceae pollen as *Erysiphe graminis* is a pathogenic fungi on species of Poaceae. The decline of pollen at the months of lower rainfall could also be due to post-anthesis and the persistence annual bush fires which usually occur during the harmattan period in South-East and North-Central Nigeria. Total pollen counts correlated negatively with rainfall, relative humidity and wind but positively with temperature. These findings are similar to those of Barnes et al. (2000), Teranishi et al. (2000), Riberio et al. (2003) and Njokuocha (2006), who found that airborne pollen concentration correlated significantly and positively with temperature and is correlated negatively with rainfall and number of rainy days. In contrast to our results, several studies obtained significant and positive correlations between daily Poaceae pollen concentration and daily maximum temperature (Valencia-Barrera et al. 2001; Green et al. 2004) or daily minimum temperature (Green et al. 2004). In this study, pollen was dominated in the month of October followed by November, and the fungal spores dominated in the month of November followed by October. This is contrary to Essien and Oluwagbemiga (2014), who found pollen more abundant in the month of May followed by June and spores more preponderant in the month of December followed by March, in the atmosphere of Anyigba, Kogi State, Nigeria. The study also was contrary to Njokuocha (2006), who found pollen more abundant in September followed by December in the atmosphere of Nsukka, Nigeria. The highly significant positive correlation of *Luffa* sp. to wind unlike the other dominant pollen indicates its pollen transport is being influenced by the prevailed local wind in the month of April and probably its localization on the trajectory of the dominant wind direction.

*Alternaria* spores were only present during the rainy season from April to September. Escuredo et al. (2011) also reported their presence in the atmosphere and their impacts on agricultural crops and human health risks. *Alternaria* spores are potential source of allergic disorders in human beings (Escuredo et al. 2011). *Alternaria solani* produces an early blight in potato crops. The pathogen can infect all aerial parts of Solanaceous crops including tomato, potato, eggplant and pepper, as well as potato tubers (Tsitsigiannis et al. 2008).

Only a significant positive correlation was observed with W (*Luffa* sp.), RH (*Hansfordiella* spp.) and significant negative correlation with T (*Puccinia* spp.). The positive correlation of 80 % of dominant fungal spores with rainfall and 90 % with relative humidity is probably related to the sporulation of fungi during the rainy season. This agrees with Lyon et al. (1984), who found significant correlation between humidity and ascospores. This finding also agrees with the report by Phanichyakarn et al. (1989), who also found dominance of fungal spores in the rainy season and lower in the dry season in the atmosphere of Bangkok. Sabariego et al. (2000) found different indices in the correlation coefficients between fungal spores concentration and meteorological parameter. From the result, 3 pollen dispersal patterns and 1 fungal season were detected:

1. Pollen morphotypes recorded in dry season and dominated by pollen from *Elaeis guineensis,* Poaceae, *Cassia* spp., *Justicia* spp., *Luffa* spp. and *Pentaclethra macrophylla*
2. Pollen morphotypes recorded during rainy season with long season (*Elaeis guineensis*, Poaceae) and short season (*Lannea acida*, *Cassia* spp.)
3. Pollen morphotypes recorded during harmattan period dominated by Poaceae, *Elaeis guineensis*, *Khaya senegalensis*, *Cyperus* spp., *Alchornea cordiforlia*, *Pentaclethra macrophylla*.
4. Rainy to late rainy season dominated by most fungal spores.

## Conclusion

This study contributes to the knowledge of the pollen and spore content of the atmosphere of Garki, Abuja. Their presence in the air was influenced by weather parameters, geography and vegetation. As a result of these variables, their atmospheric count vary from one season to the other, resulting in a more pollen load during the dry season, especially during the harmattan period and more fungal spores load during the rainy season with the exception of *Erysiphe graminis* in November. The occurrence of some dominant fungal spores could be an indicator of pathogen development in the area and could warn the farmers and agriculturists to protect their crops from diseases. We consider it necessary to include more years of sampling in order to establish correlation between total pollen and fungal spores counted and meteorological parameters.
